# The role of extracellular vesicles in the development of nasopharyngeal carcinoma and potential clinical applications

**DOI:** 10.1002/cam4.6099

**Published:** 2023-06-12

**Authors:** Tiansong Liang, Linhui Chen, Xiaojing Liu, Yuanfang Li, Zhikai Li, Daoke Yang, Huizhen Li

**Affiliations:** ^1^ Department of Radiotherapy The First Affiliated Hospital of Zhengzhou University Zhengzhou China; ^2^ School of Basic Medicine Science Zhengzhou University Zhengzhou China

**Keywords:** biomarkers, extracellular vesicles, molecular mechanisms, nasopharyngeal carcinoma, tumor therapy

## Abstract

**Background:**

Extracellular vesicles (EVs) can be secreted by a wide variety of cells, including tumor cells, and contain some bioactive molecules from the source cells. Therefore, they can potentially be used as biomarkers for early diagnosis of tumors and for tumor therapy. In addition, EVs can affect the features of target cells and participate in regulating the development process of tumors.

**Methods:**

A literature review was conducted to elucidate the role of extracellular vesicles in the progression and treatment of nasopharyngeal carcinoma.

**Results:**

In this review, we discuss the molecular mechanisms of cell proliferation, angiogenesis, epithelial‐mesenchymal transformation and metastasis, immune response, and chemo‐radiotherapy resistance that are induced by EVs. We also reviewed the potential applications of EVs as biomarkers, therapeutic agents, and carriers so as to determine new directions for the early diagnosis and targeted therapy of nasopharyngeal carcinoma. The application limitations have also been discussed in this review, further work is needed to ensure optimal outcomes for patients.

**Conclusion:**

Although the roles of extracellular vesicles in the progression of nasopharyngeal carcinoma have been summarized, some aspects are still unclear and need to be further studied. In addition, the applications of extracellular vesicles in the treatment of nasopharyngeal carcinoma still need to optimize conditions to produce better therapeutic outcomes for patients with nasopharyngeal carcinoma.

## Graphical abstract

We discuss the molecular mechanisms of cell proliferation, angiogenesis, epithelial‐mesenchymal transformation and metastasis, immune response and chemo‐radiotherapy resistance that are induced by extracellular vesicles. We also reviewed the potential applications of extracellular vesicles as biomarkers, therapeutic agents, and carriers so as to determine new directions for the early diagnosis and targeted therapy of nasopharyngeal carcinoma.

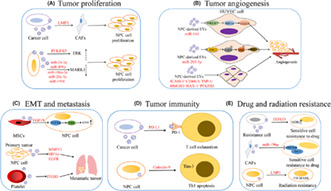

## 1 INTRODUCTION

Nasopharyngeal carcinoma (NPC), a malignant tumor originating from the nasopharyngeal epithelium, has a high incidence in southeast and east Asia, including south China.^1^, ^2^, ^3^ The major factors underlying NPC pathogenesis include infection by the Epstein–Barr virus (EBV), environmental factors and genetic susceptibility.^4^ During EBV‐latent infection, several products were released, including nuclear proteins (EBNA‐1, ‐2, ‐3, and ‐LP), membrane proteins (LMP‐1, ‐2), and non‐coding RNAs. LMP1, particularly, is a known major oncogene owing to its high expression in EBV‐associated cancers and the transforming capacity in cell lines.^5^, ^6^ Radiotherapy has become the main treatment method for non‐metastatic NPC, due to the sensitivity of NPC cells to radiation.^7^ However, treatment failure often occurs because NPC is usually first diagnosed as locoregional advanced disease due to the deep anatomical location of the tumor and lack of clinical signs, ability of NPC to infiltrate surrounding tissues and strong epithelial‐mesenchymal transformation (EMT) capability.^8^ For these reasons, it is necessary to develop novel methods for the diagnosis of early‐stage NPC and find new effective methods for treatment.

**FIGURE 1:**
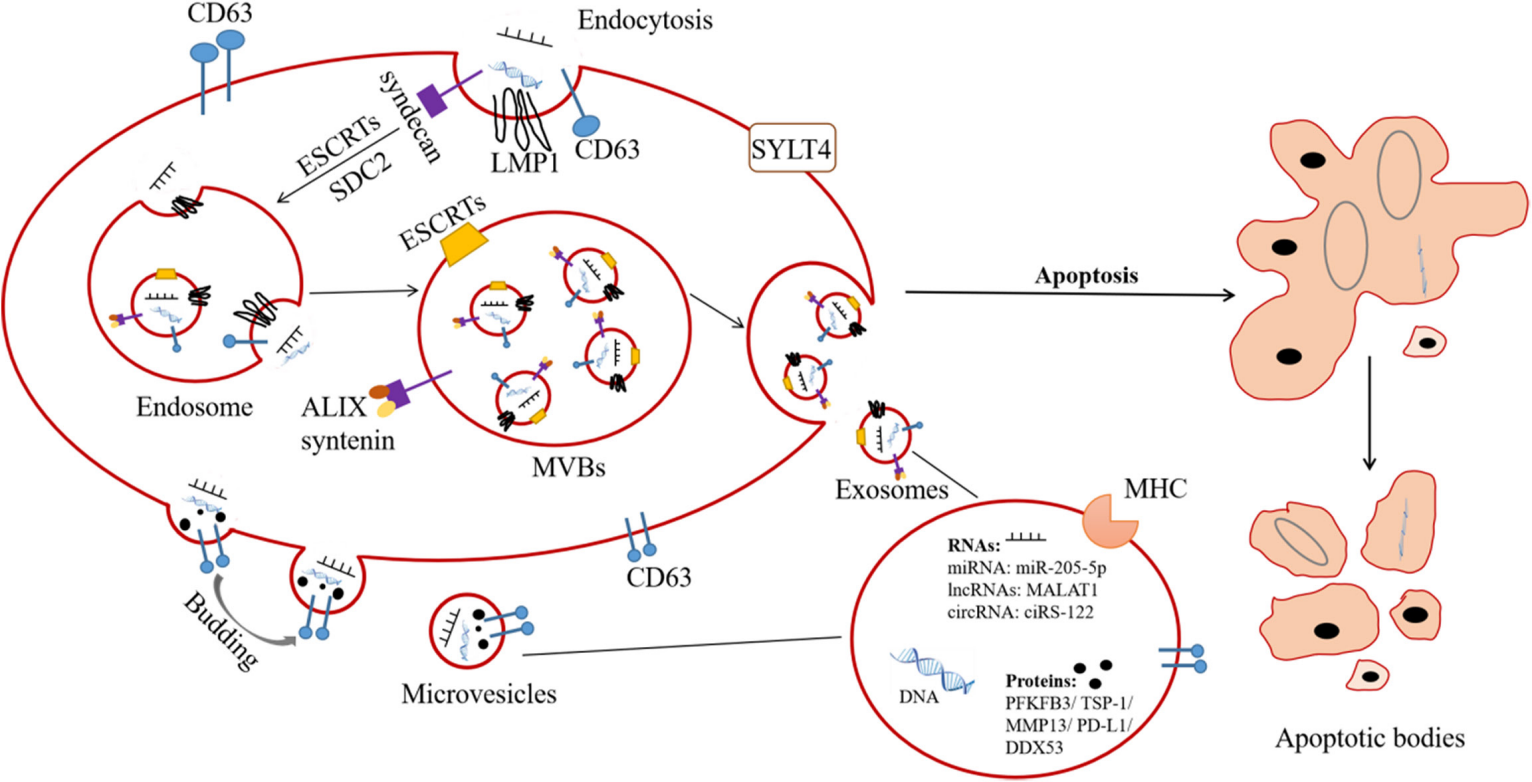
The formation and release of EVs. Exosomes (40–100 nm) are released by MVBs through fusion with the plasma membrane. Microvesicles (100–1000 nm) are released via budding from the cell membrane. Apoptotic bodies (500–4000 nm) are membrane structures released by cells during apoptosis. These EVs are composited with proteins, lipids, and nucleic acids. circRNA, circular RNA; lncRNA, long non‐coding RNA, MHC, major histocompatibility complex; MVBs, multivesicular bodies; SDC2, syndecan‐2; SYLT4, synaptotagmin‐like‐4.

In recent years, extracellular vesicles (EVs) have been found to promote the development of tumors in several different ways, and have attracted extensive attention as biomarkers and nanocarriers for diagnosis and treatment of cancer. EVs are membrane vesicles composed of a phospholipid bilayer which can be secreted by many kinds of cells, including cancer cells.^9^ They play an important role in cellular communication by transferring proteins, lipids, and nucleic acids (DNA, mRNA, and non‐coding RNAs) (Figure 1). More specifically, EVs can impact the function of target cells by transferring these biomolecules or by changing the tumor microenvironment.^10^, ^11^ Tumor‐derived extracellular vesicles (TEVs) can also carry cargoes reflecting genetic or signaling alterations of parent cancer cells to target cells, and play critical roles in regulating gene expression, cancer development, and progression.^7^, ^12^ Therefore, TEVs and their cargoes could be recognized as prospective cancer biomarkers for cancer diagnosis and prognosis. On the other hand, EVs play large and important roles in the field of tumor treatment due to their low immunogenicity and good biocompatibility, as well as high loading and delivery abilities.^13^ In this review, the pivotal roles of EVs in regulating NPC development, angiogenesis, metastasis, immune escape, and chemo‐radiotherapy resistance are discussed. In addition, we summarize and update the potential applications of EVs in the early diagnosis and treatment of NPC. We hope to provide some guidance on the roles of EVs in the pathogenesis of NPC and their clinical applications.

## 2 EVS AFFECT VARIOUS PROCESSES OF NPC

**FIGURE 2:**
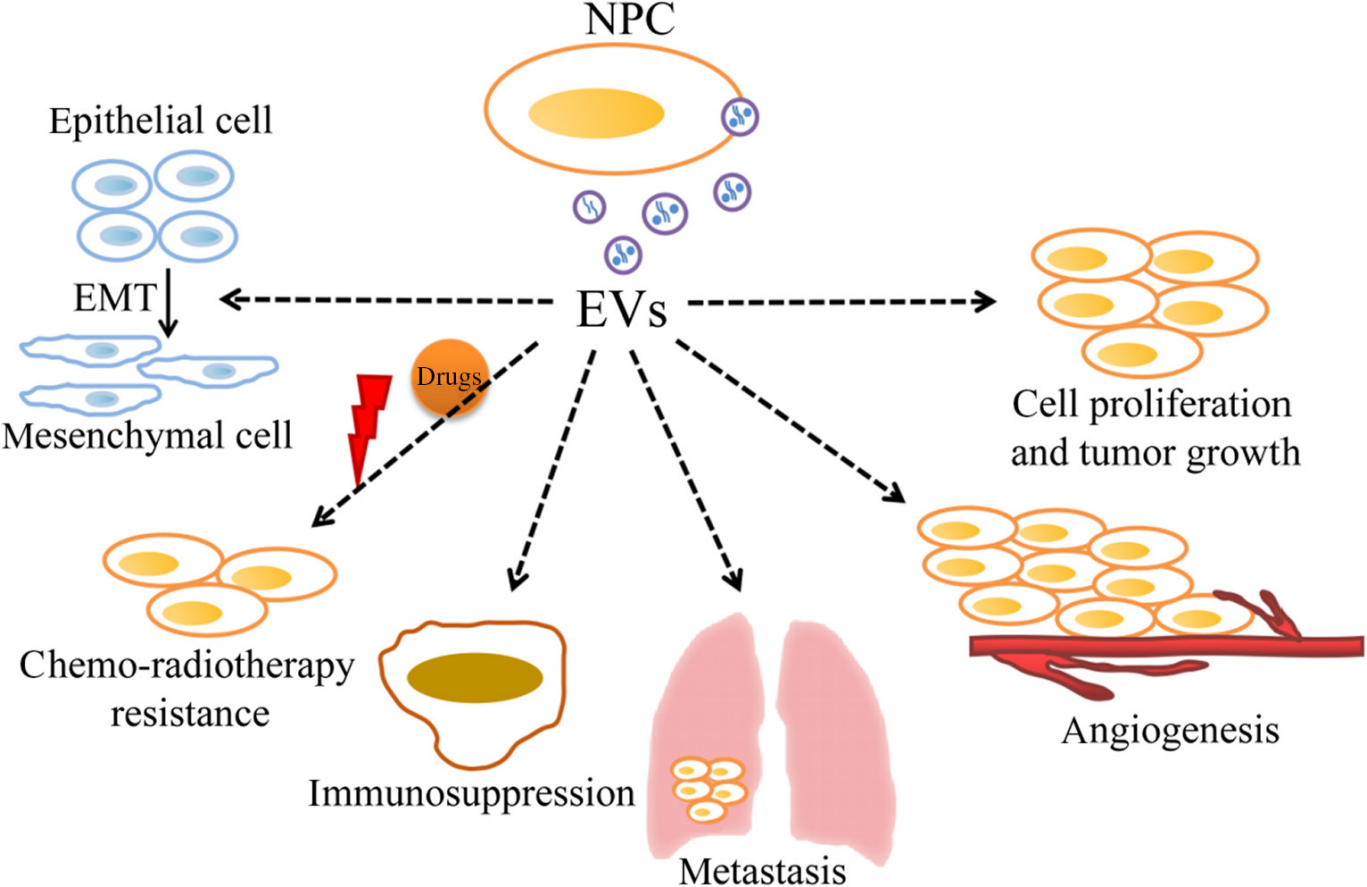
Role of EVs in the progression of NPC. EVs are involved in cell proliferation and tumor growth, angiogenesis, EMT and metastasis, immune response, and chemo‐radiotherapy resistance.

According to their origin, diameter and surface proteins, EVs can be divided into three types: exosomes, microvesicles, and apoptotic bodies; the first two types have been more extensively researched.^9^ Exosomes (40–100 nm) are released by the fusion of multivesicular bodies (MVBs) with the plasma membrane. CD63, CD9, and endosomal‐sorting complexes (ESCRTs) play important roles in the biogenesis of exosomes; in addition, the syndecan‐syntenin‐ALIX pathway is involved in the formation of exosomes.^5^ Microvesicles are vesicles with 100–1000 nm diameter that may bud off from the cell membrane.^14^, ^15^ The diverse molecular rearrangements in the plasma membrane forms the basis of biogenesis of microvesicles.^5^ Apoptotic bodies (500–4000 nm) are membrane structures released by cells during apoptosis. Breakage of apoptotic cells into apoptotic bodies initiates with condensation of the nuclear chromatin, followed by cellular morphological changes which are mainly composed of membrane blebbing (Figure 1).^16^, ^17^ Past studies have revealed that ESCRT‐III and CD63 are necessary for effectively packaging and the secretion of LMP1 into EVs, while LMP1 can induce the secretion of EVs by Hrs, syntenin‐1, and specific ESCRT‐III subunits (such as CHMP4B, CHMP5, and CHMP6) or promote EVs secretion by upregulating syndecan‐2 (SDC2) and synaptotagmin‐like‐4 (SYTL4) through NF‐κB signaling in NPC cells.^18^, ^19^, ^20^ After being released into the extracellular environment these EVs mainly exist in body fluids such as blood, urine, and breast milk, and samples of them are easily obtained.^21^ EVs in body fluids can be extracted and purified by ultracentrifugation, size‐based separation techniques, coprecipitation, and immunoaffinity enrichment.^22^ Ultracentrifugation is the gold standard for separating EVs. In addition, multiple studies have shown that EVs play essential roles in accelerating tumor growth, mediating angiogenesis, promoting EMT, and metastasis, regulating the tumor microenvironment, participating in the immune response, and inducing chemo‐radiotherapy resistance, all of which can promote the occurrence and development of NPC (Figure 2).

### 2.1 Effects of NPC‐related EVs on cell growth

Several proteins and nucleic acids encapsulated in EVs promote NPC growth. Latent membrane protein 1 (LMP1) is mainly produced in EBV infection, and it can promote cell proliferation and protect NPC cells from apoptosis.^23^ LMP1 can be packaged into EVs which then activates normal fibroblasts to become cancer‐related fibroblasts via the nuclear factor (NF)‐κB p65 signaling pathway. Autophagy and metabolic status transformation of cancer‐related fibroblasts facilitate the proliferation, migration, and radiation resistance of NPC cells.^24^ PFKFB3 (gene that encodes 6‐phosphofructo‐2‐kinase/fructose‐2,6‐biphosphatase 3), a glycolytic activator, is highly expressed in NPC‐derived EVs and facilitates cell growth, migration, and angiogenesis in NPC by influencing the cell cycle and EMT. It also reduces apoptosis by activating ERK and p‐AKT.^25^ Ye et al. found that the microRNAs (miRNAs) miR‐24‐3p, miR‐891a, miR‐106a‐5p, miR‐20a‐5p, and miR‐1908 are over‐expressed in EVs extracted from patient sera or NPC cells, and by down‐regulating microtubule affinity regulating kinase 1 (MARK1) signaling these miRNAs can affect cell proliferation and differentiation.^26^ Cell proliferation is a complex process, and EVs can affect cell proliferation through different pathways. Therefore, using EVs as targets for tumor therapy can lead to inhibition of cell proliferation through these pathways.

### 2.2 NPC‐related EVs mediate angiogenesis

Blood vessels are necessary in every part of the body to deliver oxygen and nutrients to normal cells.^27^ Angiogenesis is the process of forming new blood vessels, but these new blood vessels can also promote tumor growth and metastasis and may lead to poor prognosis.^28^ Consequently, inhibition of tumor blood vessels has become an important means for inhibiting tumor growth. However, anti‐angiogenesis therapy has failed to achieve the expected clinical effect. Nucleic acids and proteins are delivered between cells by EVs and play a crucial role in tumor angiogenesis and resistance to anti‐angiogenesis drugs.^29^ Tian and colleagues showed that miR‐144 is highly enriched in NPC‐derived EVs. It can stimulate angiogenesis by inhibiting the target gene FBXW7 (encodes F‐box/WD repeat‐containing protein 7) and promoting the expression of hypoxia‐inducible factor‐1α (HIF1α)‐dependent vascular endothelial growth factor‐A (VEGF‐A), and then ultimately enhances the metastasis of human umbilical vein endothelial cells (HUVECs).^10^ Similarly, miR‐23a from NPC‐derived EVs promotes endothelial cell migration and generation by targeting testis‐specific gene antigen 10 (TSGA10) which can accelerate angiogenesis.^30^ miR‐205‐5p in EVs promotes angiogenesis and metastasis by targeting the DSC2 gene (encodes desmocollin‐2) to enhance the EGFR/ERK signaling pathway and the expression of the matrix metalloproteinase genes MMP2/MMP9.^31^ Expect for miRNAs, EVs circRNAs can affect tumor angiogenesis by acting as miRNA and RNA‐binding protein “sponges” and by interacting with epigenetic regulators to induce cancer progression. In addition, EVs circRNAs can modulate the permeability of endothelial cells, thereby facilitating the metastasis of cancer cells.^32^ In conclusion, various RNAs in EVs play important regulatory roles in angiogenesis by targeting different molecules.^33^

Moreover, tumor angiogenesis has been shown to be mediated by many proteins from NPC‐derived EVs. Proteomic analysis shows that proteins related to angiogenesis, for example, the expression of intercellular adhesion molecule‐1 (ICAM‐1) and CD44 variant isoform 5 (CD44v5) in NPC‐derived EVs is upregulated, while that of thrombospondin‐1 (TSP‐1), an angio‐suppressive protein, is downregulated. These EVs promote angiogenesis and increase the migration and metastasis of tumor cells, as expected.^34^ High mobility group box 3 (HMGB3)‐containing nuclear exosomes secreted by NPC cells accelerate tumor metastasis by inducing angiogenesis.^35^ PFKFB3 and hematopoietic cell‐specific protein 1 (HS1)‐associated protein X‐1 (HAX‐1) can promote the growth of NPC by being transferred via EVs to recipient HUVECs, sequentially increasing proliferation, migration, and angiogenesis.^25^, ^36^ These studies demonstrate the important roles of EVs in tumor angiogenesis, and provide new targets for anti‐angiogenesis which are conducive to the development of new anti‐angiogenesis drugs.

On the other hand, bevacizumab from tumor‐derived EVs cannot bind to VEGF‐A which may be due to VEGF‐A shedding from the surface of these EVs that can induce the escape of tumor cells from treatment by bevacizumab. The study of these mechanisms may provide solutions to the problem of anti‐angiogenesis drug resistance caused by EVs, which will then be beneficial for the development of clinical drugs.^37^

### 2.3 EVs promote EMT and metastasis

NPC is prone to infiltrate the surrounding tissue space and has strong EMT ability, which can promote tumor invasion and metastasis and often lead to treatment failure.^38^, ^39^, ^40^ In the tumor microenvironment, tumors release many substances including EVs and cytokines, which can not only promote the proliferation of tumor cells but can also promote distant metastases.^41^ Predominant types of distant metastasis of tumors include lymphatic and hematogenous metastases, platelets are involved in multiple steps of hematogenous metastasis, platelet extracellular vesicles (PEVs) are the most numerous EVs in blood. Many studies have shown that PEVs could promote metastasis formation. Li et al. found that PEVs from NPC patients induced the metastasis of NPC cells by upregulating ITGB3 expression. Upregulating ITGB3 increased SLC7A11 expression by activating the MAPK/ERK/ATF4/Nrf2 axis and enhancing protein stability, that suppressed ferroptosis, therefore promoting the distant metastasis of NPC cells.^42^ Many other studies also shown that PEVs could transfer to target cells and their cargoes promoted phenotypic changes and novel functions of target cells which influenced the progression of tumor.^43^ These findings may be helpful in promoting the applications of platelet‐targeted drugs in patients with tumor.

**FIGURE 3:**
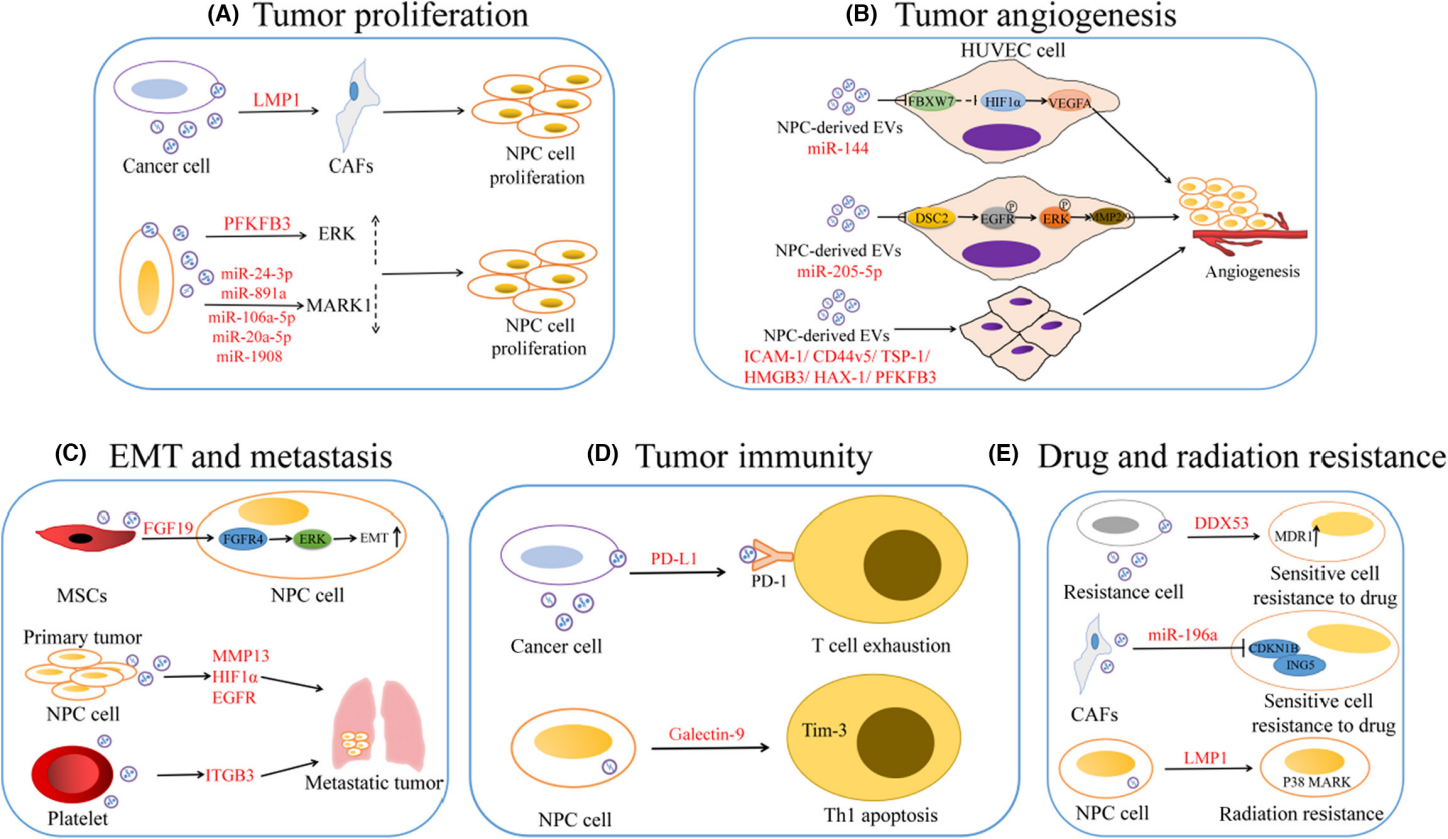
EV‐related molecules and their pathways involved in the development of NPC. CAFs, cancer‐related fibroblasts; DSC2, desmocollin‐2; PD‐1, programmed death‐1.

The interaction between the tumor microenvironment and tumor cells is influenced by EVs. Mesenchymal stem cell (MSC)‐derived EVs highly express fibroblast growth factor 19 (FGF19) which promotes NPC cell proliferation and metastasis through activating FGF19‐FGFR4‐dependent ERK signaling and by regulating EMT. One study showed that EVs participate in the progression of NPC.^44^ On the other hand, several researches have shown that EVs secreted by tumor cells are critical factors in tumor metastasis. You et al. found that EVs derived from the human NPC cell line CNE2 contained higher levels of MMP13 which significantly alters the expression of EMT markers (E‐, N‐cadherin and vimentin) and promotes the metastatic properties of NPC cells.^45^ Aga et al. demonstrated that HIF1α can be detected in EVs and that LMP1 significantly increases the expression levels of HIF1α in EVs. Importantly, HIF1α induces the alteration of EMT markers, such as E‐ and N‐cadherins, and participates in EV‐mediated pro‐metastatic effects in target cells.^46^ NPC cells with highly metastatic properties express high levels of epidermal growth factor receptor (EGFR) in EVs. In addition, in poorly metastatic cells the EGFR‐rich EVs promote EGFR upregulation and mediate reactive oxygen species (ROS) downregulation through phosphoinositide 3‐kinase and AKT (PI3K/AKT) signaling.^47^ Taken together, EVs can influence the development of NPC via different pathways (Figure 3).

### 2.4 Function of NPC‐related EVs in the immune response

In the treatment of tumors, immunotherapy is becoming increasingly important, but the effect of immunotherapeutic drugs on many patients is not ideal. A major barrier for immunotherapy is that tumor cells escape from immune surveillance.^48^ Several immune‐escape mechanisms have been described so far, including antigenic deletion, tumor cell leakage, immune inhibition caused by tumor cells, the lack of costimulatory signals on the tumor cell surface, and the anti‐apoptotic effects of tumor cells.^49^ T cells are involved in all aspects of the body's immune response, and their function and development can influence the effectiveness of immunotherapy.^50^ Tumor cells inhibit the function of T cells through different pathways. In recent years, there has been growing evidence that EVs secreted by tumor cells facilitate immune escape through inhibiting the development and function of T cells.^50^, ^51^ For example, tumor cells release EVs containing programmed death‐ligand 1 (PD‐L1, expressed on cancer cells) on their surface, and these EVs inhibit the function of CD8 T cells by binding to programmed death‐1 (PD‐1, expressed on immune cells) on T cells and promote the immune escape of tumor cells.^52^ Ye et al. found that NPC‐derived EVs highly express miR‐24‐3p. By targeting fibroblast growth factor 11 (FGF11), miR‐24‐3p impedes the proliferation of T‐cells and Th1 and Th17 differentiation. Whereas miR‐24‐3p induces differentiation of regulatory T cells, on the other hand levels of interleukin‐1β (IL‐1β), IL‐6, and IL‐10 released from CD4^+^ and CD8^+^ T cells are increased and the levels of interferon‐γ (INF‐γ), IL‐2 and IL‐17 are decreased when stimulated with NPC‐derived EVs, and then tumor pathogenesis is promoted.^26^, ^53^ One study has shown that EBV‐associated NPC cells release EVs containing high amounts of galectin‐9 which binds with T‐cell immunoglobulin domain and mucin domain 3 (Tim‐3); these EVs induce Th1 lymphocytes apoptosis. Anti‐Tim‐3 and anti‐galectin‐9 antibodies block the interaction of galectin‐9/Tim‐3 resulting in a significant decrease in the suppression of Th1 cells induced by NPC EVs. These antibodies sustain T‐cell immune responses.^54^ In addition to immunosuppression, tumor‐derived EVs can also play an immune‐stimulating role. These EVs can deliver markers of tumor cells to immune cells, facilitating the immune response. For instance, tumor‐derived EVs convey miR‐155 into dendritic cells (DCs) and promote the maturation of DCs which can then activate and proliferate lymphocytes, thereby inducing the anti‐tumor response of lymphocytes.^55^ In sum, tumor‐derived EVs can affect the function of lymphocytes and impact immune response.

At present, many immunotherapy drugs, such as immune checkpoint inhibitors are available that work by inhibiting the combination of tumor cells and immune cells, so as to restore the killing function of immune cells. Therefore, whether EVs can achieve the same effect remains to be further studied. EVs are a “double‐edged sword,” and we need to fully exploit their advantages to produce greater anti‐tumor effects.

### 2.5 EVs induce chemo‐radiotherapy resistance

Chemotherapy and radiotherapy are the most commonly used methods to treat NPC, but tumor resistance to chemo‐radiotherapy leads to poor therapeutic effect. Therefore, it is important to understand the principle of chemo‐radiotherapy resistance to achieve a better therapeutic outcome.^56^ It has been demonstrated that EVs are involved in the development of this resistance. Xiong et al. found there are four upregulated long non‐coding RNAs (lncRNAs: MALAT1, FAM212B‐AS1, LINCPINT, and H19) and seven downregulated expressed lncRNAs (SNHG16, CDKN2B‐AS1, ZFAS1, CCAT1, SNHG6, GAPLINC, and TUG1) that are associated with chemo‐radiotherapy resistance; these lncRNAs are exceptionally useful for the migration and invasion of target NPC cells when expressed by EVs derived from chemo‐radioresistant cells.^57^ DEAD‐box helicase 53 (DDX53) is over‐expressed in EVs secreted from paclitaxel‐resistant NPC cells which can be transferred into normal NPC cells via EVs. The transferred DDX53 can upregulate the level of multidrug resistance protein 1 (MDR1) in target cells to promote resistance to paclitaxel.^58^ Huang and colleagues found that EVs derived from doxorubicin‐resistant human microvascular endothelial cells facilitate proliferation, migration, EMT, and chemoresistance of co‐incubated NPC cells.^59^ The miR‐196a in EVs derived from cancer‐associated fibroblasts (CAFs) promotes proliferation and resistance to cisplatin in head and neck cancer cells by targeting the tumor suppressors cyclin‐dependent kinase inhibitor 1B (CDKN1B) and inhibitor of growth protein 5 (ING5).^60^ Tumor and other cells in the TME can secrete chemoresistant EVs containing circulating exosomal circular RNA (circRNA) that can facilitate resistance and tumor recurrence. Therefore, the roles of EVs circRNAs in the chemoresistance can lead to the development of novel therapeutic targets as more effective therapeutic agents for tumor treatment.^32^

It has been reported that circRNA is an oncogene in NPC cells which is associated with lymph node metastasis, disease recurrence and TNM stage, and can also enhance the radiotherapy resistance of tumor cells.^61^ LMP1 can promote the secretion of LMP1‐positive EVs, and these EVs can induce the proliferation and invasion of recipient NPC cells. LMP1 in EVs also stimulates radiation resistance via activating the P38 MARK signal.^62^ The mechanisms of chemo‐radiotherapy resistance induced by EVs provide new ideas for us to develop novel methods for overcoming the resistance. Perhaps EVs can also be developed as a tool to inhibit chemo‐radiotherapy resistance. As examples, either by inhibiting the synthesis or secretion of EVs or by loading chemotherapy and radiotherapy sensitizers into the EVs.

Aside from promoting tumor development and progression (Table 1), EVs can also present new possibilities for tumor treatment and can serve as potential targets for NPC treatment.

**TABLE 1:** Summary of the EVs content and their roles in NPC.

Function		Regulatory mechanism	References
Proliferation	LMP1	Activates normal fibroblasts to become cancer‐related fibroblasts	^24^
	PFKFB3	Influencing the cell cycle and EMT	^25^
	miR‐24‐3p, miR‐891a, miR‐106a‐5p, miR‐20a‐5p, miR‐1908	Down‐regulating MARK1 signaling	^26^
Angiogenesis	miR‐144	Inhibiting the target gene FBXW7 and promoting the expression of VEGF‐A	^10^
	miR‐23a	Targeting TSGA10	^30^
	miR‐205‐5p	Enhancing the EGFR/ERK signaling pathway	^31^
	ICAM‐1/CD44v5/TSP‐1/HMGB3/HAX‐1/PFKFB3	Promoting the proliferation of HUVECs	34, 35, 36
EMT and metastasis	ITGB3	Upregulating ITGB3 expression and inhibiting ferroptosis in NPC cells by upregulating SLC7A11 expression	^42^
	FGF19	Activating FGF19‐FGFR4‐dependent ERK signaling	^44^
	MMP13, HIF1α, EGFR	Altering the expression of EMT markers and promotes the metastatic properties of NPC cells	45, 46, 47
Immune response	PD‐L1	Inhibiting the function of CD8 T cells	^52^
	miR‐24‐3p	Impeding the proliferation of T‐cells, Th1 and Th17 differentiation by targeting FGF11	^53^
	galectin‐9	Inducing Th1 lymphocytes apoptosis	^54^
	miR‐155	Promoting the maturation of DCs	^55^
Chemoresistance	DDX53	Up‐regulating the level of MDR1	^58^
	miR‐196a	Targeting the tumor suppressors CDKN1B and ING5	^60^
Radioresistance	LMP1	Activating the P38 MARK signal	^62^

Abbreviations: CD44v5, CD44 variant isoform 5; CDKN1B, cyclin‐dependent kinase inhibitor 1B; DCs, dendritic cells; DDX53, DEAD‐box helicase 53; EGFR, epidermal growth factor receptor; EMT, epithelial‐mesenchymal transformation; ERK, extracellular signal‐regulated kinase; FBXW7, F‐box/WD repeat‐containing protein 7; FGF19, fibroblast growth factor 19; FGFR4, fibroblast growth factor receptor 4; HAX‐1, hematopoietic cell‐specific protein 1‐associated protein X‐1; HIF1α, hypoxia‐inducible factor‐1α; HMGB3, high‐mobility group box 3; HUVECs, human umbilical vein endothelial cells; ICAM‐1, intercellular adhesion molecule‐1; ING5, inhibitor of growth protein 5; ITGB3, integrin β3; LMP1, latent membrane protein 1; MARK, microtubule affinity regulating kinase; MDR1, multidrug resistance protein 1; miR, microRNA; MMP13, matrix metalloproteinases 13; NPC, nasopharyngeal carcinoma; PD‐L1, programmed death‐ligand 1; TSGA10, testis‐specific gene antigen 10; TSP‐1, thrombospondin‐1; VEGF‐A, vascular endothelial growth factor‐A.

## 3 POTENTIAL APPLICATIONS OF EVS IN NPC

### 3.1 Applications of EVs in the screening and diagnosis of NPC

NPC is usually a locally advanced disease by the time it is first diagnosed due to several factors: high metastatic ability, deep location of NPC in the region of the throat behind the nose and lack of obvious symptoms at an early stage.^63^ Therefore, early screening for NPC is of great importance for disease control and overall survival. In primary NPC, nasopharyngeal endoscopy with a definitive biopsy is the traditional diagnostic procedure, and it is the gold standard for diagnosis. However, nasopharyngeal endoscopy is invasive and is used in cases of suspected lesions, making it unsuitable for early detection.^64^, ^65^ Imaging modalities for NPC typically include MRI or PET‐CT. While they can be used for accurate detection of residual/locally recurrent NPC, there is a possibility that these methods will fail because of changes in the nasopharyngeal area following radiation or because imaging may be blocked by secretions and crusting.^66^ With the development of detecting technology, liquid biopsy offers promise for early cancer diagnosis in the application research of precision oncology, mainly by detecting free circulating tumor cells (CTCs) or circulating tumor DNA (ctDNA) fragments or EVs in body fluids. The key to liquid biopsy success is finding specific tumor markers.

Biomarker is a quantifiable biological indicator of specific biological state that should be (i) a mediator of the disease pathology, (ii) present a stable expression level in healthy individuals and an abnormal expression level in patients, and (iii) simple and quick to evaluate.^67^ Biomarkers can be used in multiple ways as tools for (i) differential diagnosis of cancer, (ii) objective indicators of disease prognosis or staging, or (iii) prediction and management of clinical response to an intervention.^68^, ^69^ Therefore, it is important for the identification and quantification of biomarkers. Proteins are the functional molecules in an organism and may be affected by diseases, and proteomics holds a special promise in detecting pathological conditions. A variety of mass spectrometry (MS)‐based proteomic methods have been applied for the identification and quantification of proteins in biological and clinical samples to obtain biomarker candidates. However, there are still significant challenges to realize the diagnostic and prognostic potential of these biomarkers in clinical practice. Moreover, efforts are needed to improve the MS technologies to explore lower abundance proteins.^67^ The detection for NPC by circulating biomarkers, such as EBV‐specific IgA antibody testing by ELISA assays are only semi‐quantitative and lack both sensitivity and specificity.^2^, ^69^ Plasma EBV DNA has been used as a diagnostic biomarker for NPC, but its sensitivity for Stage I NPC is controversial and not all NPC cases are associated with EBV.^70^ Therefore, new biomarkers that can be used for early diagnosis and prognosis of NPC need to be found. In recent years, EVs have become a new source of biomarkers in liquid biopsy, and have attracted the extensive attention of researchers. EVs can be secreted by a variety of cells, including tumor cells. Tumor‐derived EVs can be used as biomarkers because the cargoes they carry reflect the characteristics of tumor cells, and the cargoes are frequently different from those carried by healthy‐derived EVs.^71^ EVs can protect these cargoes from degradation,^7^ they are easily obtained from several types of body fluids that can be collected through non‐invasive or minimally invasive methods.^71^ On the other hand, EVs can be used as biomarkers for detecting early stage asymptomatic cancers and for monitoring tumor progression.^72^

Cyclophilin A (CYPA) is a primary cytosolic binding protein of the immunosuppressive drug cyclosporin A. CYPA is involved in a complex interplay of proteins and signal pathways that is associated with cancers.^73^ Immunoserological markers such as IgA antibodies against EBV (EBV‐VCA‐IgA) have shown to be used for early diagnosis.^74^ However, the positive detection rate usually reaches no more than 70% in NPC patients, and thus cannot meet the clinical need. Liu et al. found that among the EBV‐VCA‐IgA negative cases, over 80% of them contained more exosomal CYPA than the standard. They found that the use of both exosomal CYPA and EBV‐VCA‐IgA to detect EBV‐associated NPC had higher discriminatory ability than use of EBV‐VCA‐IgA detection alone.^75^ In short, their study showed that exosomal CYPA is a novel potential biomarker of NPC, especially when the level of EBV‐VCA‐IgA is below the standard.

In addition, several studies have found that the diagnosis, progression, and prognosis of certain cancers can be detected by many TEV protein biomarkers.^76^, ^77^ However, the extremely low expression of some biomarkers on TEVs makes their detection by some methods difficult, such as ELISA, western blot, or flow cytometry. In recent years, the development of biosensors in the application of detection has attracted extensive attention. CD109 is specifically expressed in tumor tissues and is an excellent NPC biomarker, and it also selectively enriched on TEVs.^78^, ^79^ Li et al. developed an ultrasensitive method to measure CD109 and EGFR proteins on TEVs by using the aptamer‐CRISPR/Cas12a assay. This method mainly consists of three steps: aptamer recognition, PCR amplification in situ, and CRISPR/Cas12a detection. Real‐time fluorescence quantitative detection of CD109/EGFR‐apt is possible and the fluorescence intensity is directly proportional to the initial concentration of CD109^+^/EGFR^+^ TEVs. This assay has high sensitivity and specificity, and can also be used for detection in clinical samples, as an effective EVs detection method.^12^ Similarly, apta‐HCR‐CRISPR assay can also be used for the detection of NPC‐associated EV proteins, such as nucleolin and PD‐L1. This method is more sensitive than aptamer‐ELISA and apta‐HCR‐ELISA, which is useful for the quantification of TEVs proteins in clinical samples.^80^ In addition, Liu and colleagues established a method named PLA‐RPA‐TMA assay: two proximity ligation assay (PLA) probes recognize biomarkers such as LMP or EGFR protein on tumor‐derived EVs in NPC, and then generate the unique surrogate DNA signal for these biomarkers. The DNA signal is amplified twice by recombinase polymerase amplification (RPA) and transcription‐mediated amplification (TMA). The outcomes of the RPA‐TMA reaction are quantitatively detected by a colorimetric assay based on gold nanoparticles. The PLA‐RPA‐TMA assay allows TEVs to be detected with high specificity and sensitivity.^81^

In addition to proteins, RNAs in EVs have also been considered as potential tumor biomarkers. miRNAs, a form of endogenous noncoding RNA containing about 22 nucleotides, play important roles in regulation of gene expression, tumor growth, and treatment resistance.^82^ On the other hand, miRNAs are differently expressed in TEVs under pathological conditions. Therefore, TEVs and contained miRNAs can be used as novel diagnostic biomarkers. Jiang and colleagues identified circulating small EV‐derived miRNAs as biomarkers for the diagnosis of NPC. This study demonstrated that miRNAs, such as miR‐134‐5p, miR‐205‐5p, and miR‐409‐3p, derived from circulating small EVs of patients with NPC are different from the others who are not NPC patients, so these miRNAs can be used as good diagnostic biomarkers for detection and prognosis of NPC because of their excellent discriminative capabilities.^7^ All in all, EVs derived from various body fluids provide a novel technique for tumor diagnosis (Table 2).

**TABLE 2:** Clinical use of EVs as biomarkers.

Type	Sample	Biomarkers of EVs	Clinical application	References
Proteins	Serum	CYPA/EBV‐VCA‐IgA	The combination of exosomal CYPA and EBV‐VCA‐IgA increases the accuracy of NPC diagnosis, especially when EBV‐VCA‐IgA is negative	^75^
	Serum	CD109/EGFR	The combination of CD109^+^ and EGFR+ TEVs can be used for NPC diagnosis with high sensitivity and high specificity as well as for radiotherapy surveillance	^12^
	Serum	Nucleolin/PD‐L1	Nucleolin+ TEVs can be used for early NPC diagnosis, PD‐L1+ EVs can be used for monitoring the efficacy of immunotherapy	^80^
	Plasma	LMP or EGFR	LMP+ TEVs and EGFR+ TEVs can be used for the early diagnosis of NPC	^81^
RNAs	Plasma	miR‐134‐5p, miR‐205‐5p, miR‐409‐3p	miRNAs in circulating small extracellular vesicles can be used for the detection and prognosis of NPC	^7^

Abbreviations: CYPA, cyclophilin A; EBV‐VCA‐IgA, IgA antibodies against Epstein–Barr virus; EVs, extracellular vesicles; TEVs, tumor extracellular vesicles.

Emerging evidence suggests that EVs may be a promising tool to facilitate the diagnosis of cancer. However, because of the low levels of EVs in body fluids, a highly sensitivity technology is required for detection. Otherwise, a false‐negative result may be obtained. In addition, the collection and purification of EVs is a tedious and time‐consuming procedure, during which the proteins on the surface of EVs may be destroyed, giving inaccurate detection outcomes. Therefore, EVs extraction and purification technologies warrant further improvement. In addition, the EVs biomarkers reported in the studies so far should be validated in a larger number of independent patient cohorts so as to determine their specificity, sensitivity, and accuracy in cancer diagnosis to make them applicable for clinical diagnosis.

### 3.2 Applications of EVs in the therapy of NPC

With the development of nanotechnology, nanomaterials have been widely used in the field of tumor therapy. The nanomaterials, such as dendrimers,^83^ inorganic nanomaterials,^84^ micelles,^85^ virus‐like protein particles^86^ and liposomes,^87^ can improve the therapeutic effect of cargoes and reduce the toxic side effects of drugs. However, these nanomaterials have poor biocompatibility, they are easily cleared by the body and may cause immune response. As a natural carrier involved in communication between cells, EVs have attracted extensive attention in the field of tumor therapy and exhibited potential advantageous features. EVs have good biocompatibility and can cross a variety of biological barriers,^88^ and some proteins such as CD47 on the surface of EVs can help these vesicles evade clearance by mononuclear macrophages.^89^

According to the roles of EVs in the development of NPC as well as the structure and composition of EVs, their applications in NPC therapy can divided into the following aspects. First, EVs are involved in the occurrence and development of NPC, so directly targeting tumor‐associated EVs may play a part in tumor therapy. Zuo and colleagues discovered the promising therapeutic effect of aspirin in NPC treatment through its targeting of exosomal EBV‐LMP1 and modulation of miR‐203 expression. Aspirin can inhibit EMT through the functional NF‐κB/miR‐203/CDH6 axis, and observably suppressed NPC lung metastasis.^90^ However, the role of EVs is complex. Effects on EVs may affect the function of normal tissues and cells, thus resulting in side effects. Clearly, further investigations of using such EVs for treatment will require many clinical trials in the future.

Second, EVs have been developed as tumor vaccines. EVs secreted by tumor cells express and transfer a large number of tumor‐associated antigens (TAAs) to DCs and promote the occurrence of the immune response, and have been used as promising vaccines.^91^, ^92^ In addition, EVs contain richer immunostimulatory components compared with cells, and previous studies revealed that TEVs improve vaccine efficacy compared to tumor lysates.^93^, ^94^ However, TEVs may also contain potentially immunosuppressive molecules that can promote the progression of tumors. TEVs carry PD‐L1 on their surface which binds with T cells via PD‐1 on the T‐cell surface, thereby suppressing the function of T cells and facilitating tumor growth.^52^ In contrast, DC‐derived EVs are devoid of these immunosuppressive molecules because the activation state of their cellular source is under control, so they can be used as potential tumor vaccines.^92^ On the other hand, mature DC‐derived EVs bear tumor antigens that may lead to an immune response, suggesting that they can be used for immunotherapy in clinical practice.^95^ These vaccines slightly improved the overall survival in patients with early stage diseases. For patients with advanced or metastatic tumors, cancer vaccines may play a therapeutic role in combination therapy.^96^ However, difficulties such as the lack of quality control and standards for EV characterization and purification must be overcome. Moreover, manufacturing, storage, and administration of EVs‐based vaccines need to be addressed.

Third, as natural carriers, EVs have been widely used in NPC therapy due to their advantages.^97^ MSC‐derived exosomes can deliver miR‐34c which can inhibit NPC invasion, metastasis, proliferation, and EMT by impeding β‐chain protein.^98^ Wang and colleagues found that exosomes tagged with the tumor‐homing and penetrating peptide iRGD contained antagomiR‐BART10‐5p and antagomiR‐18a that can suppress the angiogenesis and proliferation of NPC cells by regulating the expression of HIF1‐α and VEGF in a Spry3‐dependent manner.^99^ And we found that EVs derived from tumor cells can improve the delivery efficiency of the cargoes which are widely used as the carriers for tumor therapy. We extracted EVs from tumor cells and used them to deliver chemotherapy drugs. The results suggested that the EVs as carriers have good targeting and delivery efficiency, and the effect of the drugs delivered by EVs were increased by dozen‐fold. Therefore, EVs are of great value as drug carriers.^100^ Zhu et al. also used tumor‐derived EVs to deliver miR‐142‐5p which can inhibit HGF/C‐MET and EGF/EGFR pathways to increase the radio‐sensitivity of NPC cells (Table 3).^101^ Although EVs have good biocompatibility and other advantages as carriers, albeit there are some challenges. EVs need external modification to increase the effectiveness of targeted delivery, but the modification methods are few, and the load rate of cargoes is low. Moreover, there are more challenges associated with large‐scale production of EVs that should be overcome to introduce EVs products into clinical practice.

**TABLE 3:** Applications of EVs as carriers.

Cargoes	Source	Effect	References
miR‐34c	MSCs	Inhibiting NPC invasion, metastasis, proliferation and EMT	^98^
AntagomiR‐BART10‐5p, antagomiR‐18a	HUVECs	Suppressing angiogenesis and growth of NPC cells	^99^
Doxorubicin, lonidamine	Tumor cells	Inhibiting cancer cell proliferation by damaging DNA, reduces ATP synthesis and accelerates ROS generation synergistically	^100^
miR‐142‐5p	Tumor cells	Increasing the radio‐sensitivity of NPC cells	^101^

Abbreviations: MSCs, mesenchymal stem cells; ROS, reactive oxygen species.

## 4 CONCLUSIONS

In brief, EVs have aroused extensive attention of researchers. EVs secreted from different NPC cells play important roles in tumor progression, and so they can also serve as potential targets for tumor therapy and provide new possibilities for the development of clinical drugs. In addition, EVs have potential applications in tumor diagnosis and treatment. They can be developed as non‐invasive biomarkers for the diagnosis of NPC, providing a possibility for early diagnosis. And because EVs can carry tumor‐associated antigens, they can be used as tumor vaccines for immune prevention. EVs have good biocompatibility and low immunogenicity, suggesting they can be used as ideal carriers for tumor therapy. Conclusively, this article reviews the roles of EVs in the pathogenesis of NPC and their potential clinical applications, and we hope to provide points of reference for future clinical interventions. However, research so far has been limited to the laboratory level, and thus the development of potential clinical applications of EVs needs to be accelerated. From our review, we consider that the following aspects still need to be improved. First, the separation and purification methods of EVs still need to be further modified to find the most efficient extraction methods. Second, loading methods of drugs need to be improved to enhance the drug loading rates. Third, new factors affecting the secretion of EVs need to be further clarified. Fourth, tumor‐unique biomarkers need to be found for tumor detection. To solve these problems, more research is needed.

## AUTHOR CONTRIBUTIONS

**Tiansong Liang:** Conceptualization (equal); writing – original draft (equal); writing – review and editing (supporting). **Linhui Chen:** Writing – review and editing (supporting). **Xiaojing Liu:** Writing – review and editing (supporting). **Yuanfang Li:** Writing – review and editing (supporting). **Zhikai Li:** Writing – review and editing (supporting). **Daoke Yang:** Conceptualization (equal). **Huizhen Li:** Conceptualization (equal); writing – original draft (equal); writing – review and editing (lead).

## FUNDING INFORMATION

Accessing the literature and writing the manuscript were supported by the First Affiliated Hospital of Zhengzhou University (LHGJ20190046).

## CONFLICT OF INTEREST STATEMENT

The authors declare that they have no competing interests.

## ETHICS STATEMENT

Not applicable.

## Data Availability

N/A.
